# SpyRing interrogation: analyzing how enzyme resilience can be achieved with phytase and distinct cyclization chemistries

**DOI:** 10.1038/srep21151

**Published:** 2016-02-10

**Authors:** Christopher Schoene, S. Paul Bennett, Mark Howarth

**Affiliations:** 1 Department of Biochemistry, University of Oxford, South Parks Road, Oxford, OX1 3QU, UK; 2 Sekisui Diagnostics UK Ltd., Operations Building, Liphook Way, Allington, Maidstone, Kent, ME16 0LQ, UK

## Abstract

Enzymes catalyze reactions with exceptional selectivity and rate acceleration but are often limited by instability. Towards a generic route to thermo-resilience, we established the SpyRing approach, cyclizing enzymes by sandwiching between SpyTag and SpyCatcher (peptide and protein partners which lock together via a spontaneous isopeptide bond). Here we first investigated the basis for this resilience, comparing alternative reactive peptide/protein pairs we engineered from Gram-positive bacteria. Both SnoopRing and PilinRing cyclization gave dramatic enzyme resilience, but SpyRing cyclization was the best. Differential scanning calorimetry for each ring showed that cyclization did not inhibit unfolding of the inserted β-lactamase. Cyclization conferred resilience even at 100 °C, where the cyclizing domains themselves were unfolded. Phytases hydrolyze phytic acid and improve dietary absorption of phosphate and essential metal ions, important for agriculture and with potential against human malnutrition. SpyRing phytase (PhyC) resisted aggregation and retained catalytic activity even following heating at 100 °C. In addition, SpyRing cyclization made it possible to purify phytase simply by heating the cell lysate, to drive aggregation of non-cyclized proteins. Cyclization via domains forming spontaneous isopeptide bonds is a general strategy to generate resilient enzymes and may extend the range of conditions for isolation and application of enzymes.

Purified enzymes are finding a range of important uses, from high-cost low-scale applications such as GMP-grade asparaginase in treatment of leukemia^1^, to low-cost high-scale applications such as the millions of tons per year of high fructose corn syrup biotransformed by glucose isomerase^2^. When enzymes are being developed for application in new areas, a major challenge is the stability of the enzyme to inactivation during isolation or use in its new environment. Stability can come from sourcing enzymes from harsh environments or from enzyme-specific optimization campaigns using iterative mutation^3^. However, it is also important to seek generic approaches capable of stabilizing a wide range of enzymes. One such approach is cyclization, connecting the enzyme’s N-terminus to the C-terminus. Cyclization can be accomplished via split inteins^4^,^5^,^6^,^7^, sortases^8^, carbodiimide cross-linking^9^ and, most recently, via the design of SpyRings^10^. SpyTag is a peptide tag that we engineered from *Streptococcus pyogenes* FbaB adhesion protein to form a spontaneous isopeptide bond upon binding to its protein partner SpyCatcher^11^,^12^. SpyRing cyclization utilizes SpyTag on the N-terminus and SpyCatcher on the C-terminus to cyclize an enzyme of interest. We showed this cyclization increased the thermal tolerance of TEM-1 β-lactamase (BLA) by >60 °C, both in terms of aggregation resistance and retaining activity after heating^10^. This approach to enzyme resilience is not known in nature and so it is important to further explore this phenomenon, as well as to test whether SpyRing cyclization could be extended to an industrially important class of enzymes.

At least 2 billion people are thought to suffer from deficiency of micronutrients, including vitamin A, iodine, iron, niacin, zinc and calcium^13^. Such deficiency, termed the “silent hunger”, has global consequences, including decreased bone mineralization, anemia, impaired learning, and decreased resistance to infectious diseases such as AIDS and tuberculosis^13^,^14^. Major effort has gone into increasing the nutritional value of major food crops around the world, notably introducing the β-carotene pathway into rice^15^. Most crops store phosphate as phytic acid (inositol hexakisphosphate), making the phosphate unavailable for humans as well as other agriculturally-important non-ruminants (e.g. poultry, fish, and pigs). Moreover, phytic acid is an anti-nutrient which chelates essential mineral cations (iron, zinc, calcium)^16^,^17^. Adding phytase to the diet breaks down phytic acid and has been widely used in agriculture to improve animal health^18^. The use of phytase also reduces the danger, if phosphate is directly added to feedstock, of excess phosphate polluting waterways and leading to eutrophication^18^. The thermal tolerance of phytase has great significance because feed-stock must be subjected to temperatures of 75–90 °C to kill *Salmonella* and other dietary pathogens^18^.

Here we have explored the principle of SpyRing resilience by evaluating enzyme cyclization with alternative recently-established isopeptide-forming protein chemistries. We then extended the scope of SpyRing cyclization by enhancing the thermo-resilience of a phytase. We studied the stability effects of cyclization by differential scanning calorimetry (DSC) and circular dichroism (CD). In addition we opened up a new approach for purification of SpyRing-cyclized enzymes based simply upon heating.

## Results

### Enzyme cyclization using Isopeptag/Pilin-C or SnoopTag/SnoopCatcher spontaneous isopeptide bond formation

**Figure 1:**
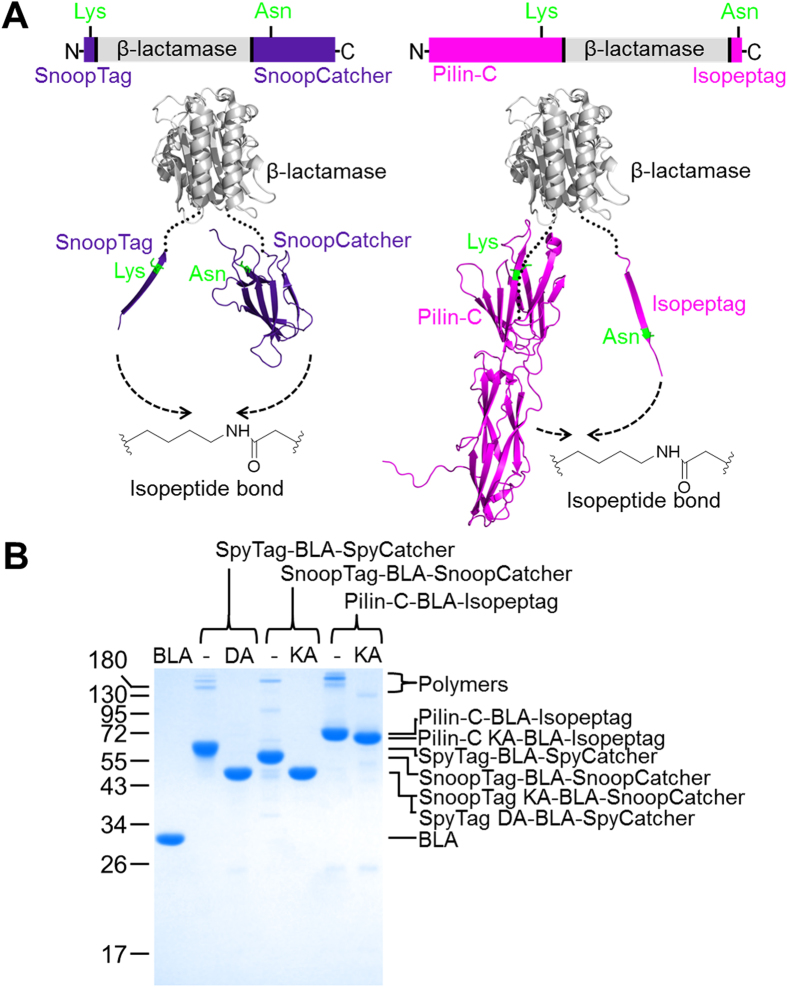
Enzyme cyclization using alternative isopeptide-forming domains. (**A**) Schematic of SnoopRing and PilinRing cyclization of β-lactamase (BLA). Proteins are shown in cartoon format, with reacting residues shown in green in stick format. (**B**) SDS-PAGE analysis of SpyRing, SnoopRing and PilinRing cyclization. Each purified protein is shown alongside the non-reactive control (DA or KA), after staining with Coomassie.

To understand the dramatic increase in thermal resilience caused by SpyRing-mediated cyclization, we compared the effects of cyclizing with two different Tag/Catcher pairs, each able to form a spontaneous isopeptide bond (Fig. 1A). First we sandwiched BLA between Isopeptag and Pilin-C, a Tag/Catcher pair we had previously engineered by splitting the *Streptococcus pyogenes* major pilin Spy0128^19^,^20^. We termed this approach PilinRing cyclization. We also tested sandwiching BLA between SnoopTag and SnoopCatcher, a peptide/protein pair we recently engineered from the RrgA adhesin of *Streptococcus pneumoniae*^21^,^22^ (SnoopRing cyclization, Fig. 1A). SpyRing cyclization occurs through reaction of an amine with a carboxylic acid with release of water, but both SnoopRing and PilinRing cyclization occur through reaction of an amine with a carboxamide with release of ammonia (Fig. 1A)^20^,^22^. Cyclization was evaluated by the change in SDS-PAGE mobility upon cyclization, compared to negative controls having the key reactive lysine mutated to alanine (SnoopTag KA-BLA-SnoopCatcher and Pilin-C KA-BLA-Isopeptag) (Fig. 1B). We found that the PilinRing system was efficient at cyclizing BLA when Pilin-C was fused to the N-terminus and Isopeptag was fused to the C-terminus (Fig. 1B). SnoopRing cyclization was efficient when fusing SnoopTag on the N-terminus and SnoopCatcher on the C-terminus (Fig. 1B). Electrospray ionization mass spectrometry (MS) confirmed the loss of ammonia for both the PilinRing and SnoopRing upon isopeptide bond formation (Fig. S1). Small amounts of high molecular weight forms were produced by both PilinRing and SnoopRing systems (but not the point mutant controls), consistent with a proportion of intermolecular reaction, as seen with SpyRing cyclization (Fig. 1B). The k_cat_ and K_M_ values of the SpyRing, SnoopRing and PilinRing cyclized BLA constructs were comparable to those of the wild-type BLA construct (Fig. S2).

### Comparison of different cyclization domains for β-lactamase resilience

**Figure 2:**
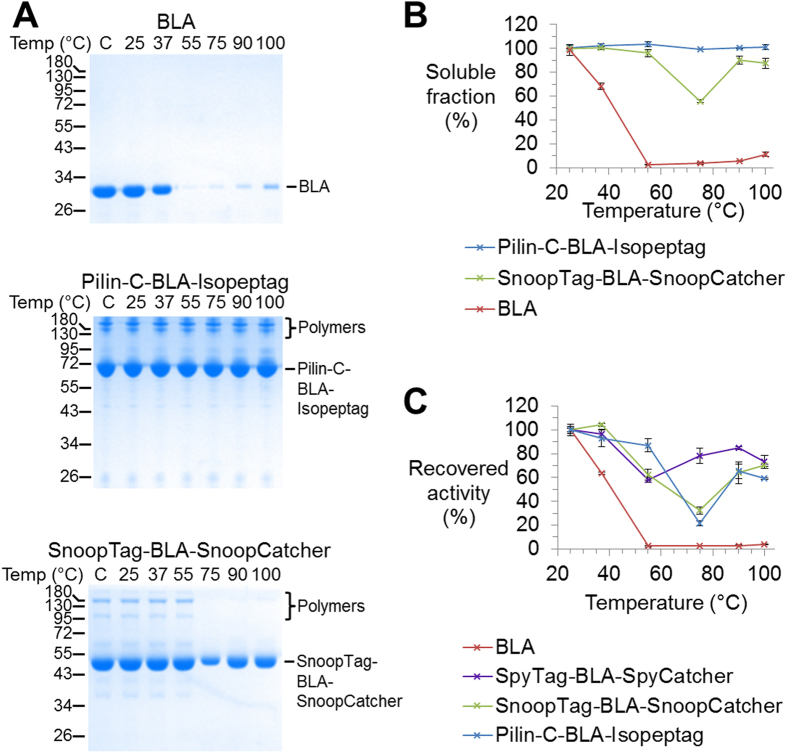
SnoopRing and PilinRing cyclization conferred thermal resilience. (**A**) Cyclization decreased heat-induced aggregation. BLA, Pilin-C-BLA-Isopeptag and SnoopTag-BLA-SnoopCatcher were heated at the indicated temperature for 10 min, centrifuged, and the supernatant analyzed by SDS-PAGE with Coomassie staining. C is control without incubation. (**B**) Plot of heat-induced aggregation, from quantifying SDS-PAGE. (**C**) Cyclization enhanced the catalytic activity following heating. BLA, SnoopTag-BLA-SnoopCatcher, Pilin-C-BLA-Isopeptag and SpyTag-BLA-SpyCatcher were incubated for 10 min at the indicated temperature, before returning to RT and then running a nitrocefin colorimetric activity assay. The initial rates of reaction were calculated using the linear portion of the enzyme curves. Data were normalized using the initial rate of reaction at 25 °C as 100% recovered activity. (All are mean of triplicate ± 1 s.d.; some error bars are too small to be visible.)

We evaluated SnoopRing and PilinRing aggregation-resistance by heating the enzymes for 10 min at various temperatures, centrifuging at high speed to remove aggregates, and analyzing the remaining soluble protein by SDS-PAGE with Coomassie staining. SpyRing was previously found to provide full aggregation-resistance to temperatures up to 100 °C^10^. For BLA itself, little protein remained in solution above 37 °C (Fig. 2A). SnoopRing and PilinRing cyclization both led to major increases in aggregation resistance (Fig. 2A,B). For Pilin-C-BLA-Isopeptag, there was little loss of soluble protein even after heating to 100 °C (Fig. 2A,B). For SnoopTag-BLA-SnoopCatcher, solubility was well preserved after heating to 100 °C, although there was more aggregation at 75 °C than at 100 °C (Fig. 2A,B).

We then tested recovery of catalytic activity after this heating step followed by cooling to room temperature. Similar to the aggregation data, nearly all catalytic activity of BLA itself was lost after 55 °C heating (Fig. 2C). SnoopRing cyclization and PilinRing cyclization greatly improved the recovery of catalytic activity, although the retention of activity was less than with SpyRing cyclization, particularly after 75 °C heating (Fig. 2C). However, Pilin-C-BLA-Isopeptag exhibited the best thermal resilience after incubation at 55 °C (Fig. 2C).

The non-cyclized control SnoopTag KA-BLA-SnoopCatcher showed little difference in aggregation-resistance or catalytic recovery compared to BLA (Fig. S3A,C). The non-cyclized control Pilin-C KA-BLA-Isopeptag did have enhanced resilience to aggregation and more retention of catalytic activity, when compared to BLA, but was clearly inferior to the cyclized PilinRing (Fig. S3B,C).

### Calorimetry of β-lactamase unfolding with alternative cyclization domains

**Figure 3:**
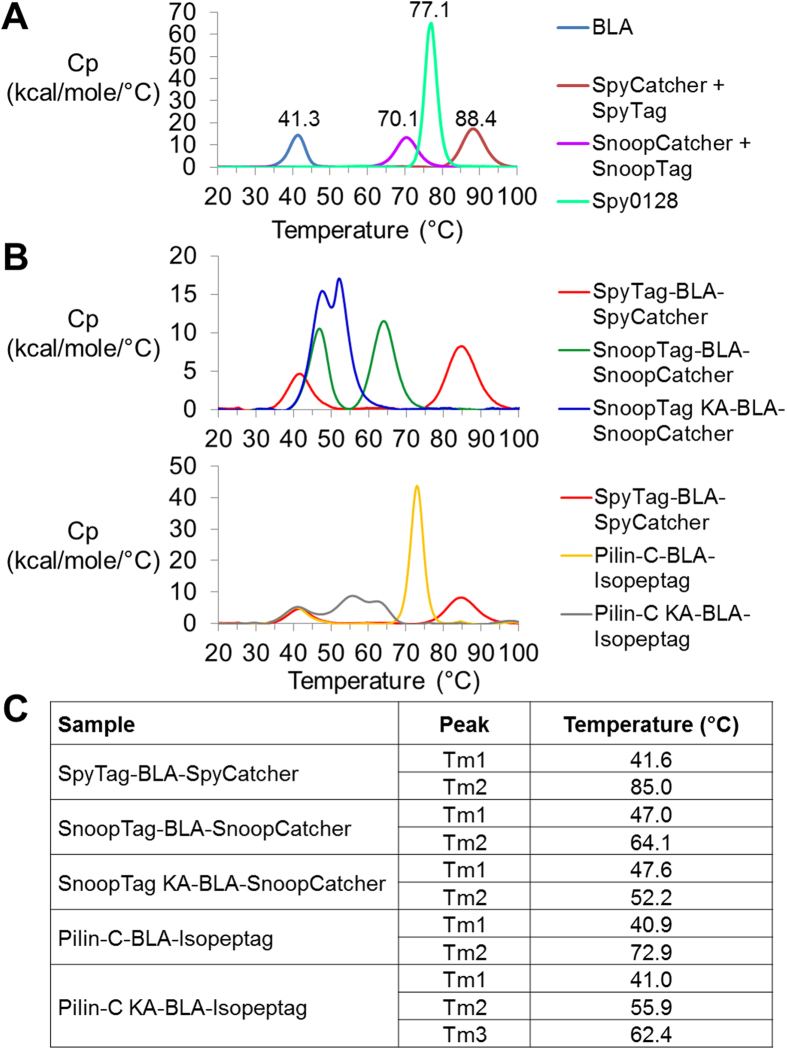
DSC comparison of SpyRing, SnoopRing and PilinRing BLA. (**A**) DSC showing the melting profiles of BLA overlaid with SpyCatcher + SpyTag peptide, SnoopCatcher + SnoopTag peptide, and Spy0128, giving the specific heat capacity (Cp) of each protein scanned from 20–100 °C at 1 °C/min. (**B**) DSC showing the melting profile of SpyTag-BLA-SpyCatcher overlaid with SnoopTag (KA)-BLA-SnoopCatcher or Pilin-C (KA)-BLA-Isopeptag, analyzed as in (A). (**C**) Tm values for peaks observed in (B).

To explore the difference in resilience for BLA cyclized by different domains, we performed DSC on SpyRing, PilinRing and SnoopRing systems. First we investigated the unfolding of the individual domains. SpyCatcher after reconstitution with SpyTag peptide unfolded at 88 °C, higher than SnoopCatcher reconstituted with SnoopTag peptide (70 °C) or Spy0128 (77 °C), the parent domain split to generate Pilin-C/Isopeptag (Fig. 3A).

We previously showed that SpyTag-BLA-SpyCatcher did not change the Tm for BLA unfolding, giving a similar peak at ~41 °C for BLA (Fig. 3A) and SpyTag-BLA-SpyCatcher^10^ (Fig. 3B,C). For SpyTag-BLA-SpyCatcher we also observed a peak from SpyTag/SpyCatcher unfolding at 85.0 °C (Fig. 3B,C)^10^,^23^. For the SnoopRing and its KA control, surprisingly there was a small increase in the BLA Tm to 47.0 °/47.6 °C (Fig. 3B,C). We suggest that the 64.1 °C peak is likely to correspond to unfolding of the SnoopTag/SnoopCatcher domain and the 52.2 °C peak to unfolding of the SnoopTag KA/SnoopCatcher domain (Fig. 3B,C).

For the PilinRing and its non-cyclized control, the Tm for BLA unfolding was ~41 °C, the same as SpyTag-BLA-SpyCatcher (Fig. 3B,C). We suggest that the 72.9 °C peak is likely to correspond to unfolding of the Pilin-C/Isopeptag domain and the 55.9 and 62.4 °C peaks to unfolding of Pilin-C KA/Isopeptag (Fig. 3B,C). Therefore, as for SpyTag/SpyCatcher, cyclization via an isopeptide bond-forming domain had no effect (PilinRing) or a minor effect (SnoopRing) on the transition temperature of the sandwiched BLA domain.

Overall, PilinRing cyclization improved enzyme resilience substantially, but gave less catalytic recovery than SpyRing cyclization. Since Pilin-C is also much larger than SpyCatcher (31 kDa rather than 12 kDa), subsequent investigation focused on advancing and understanding SpyRing cyclization.

### SpyRing cyclization made phytase thermo-resilient

**Figure 4:**
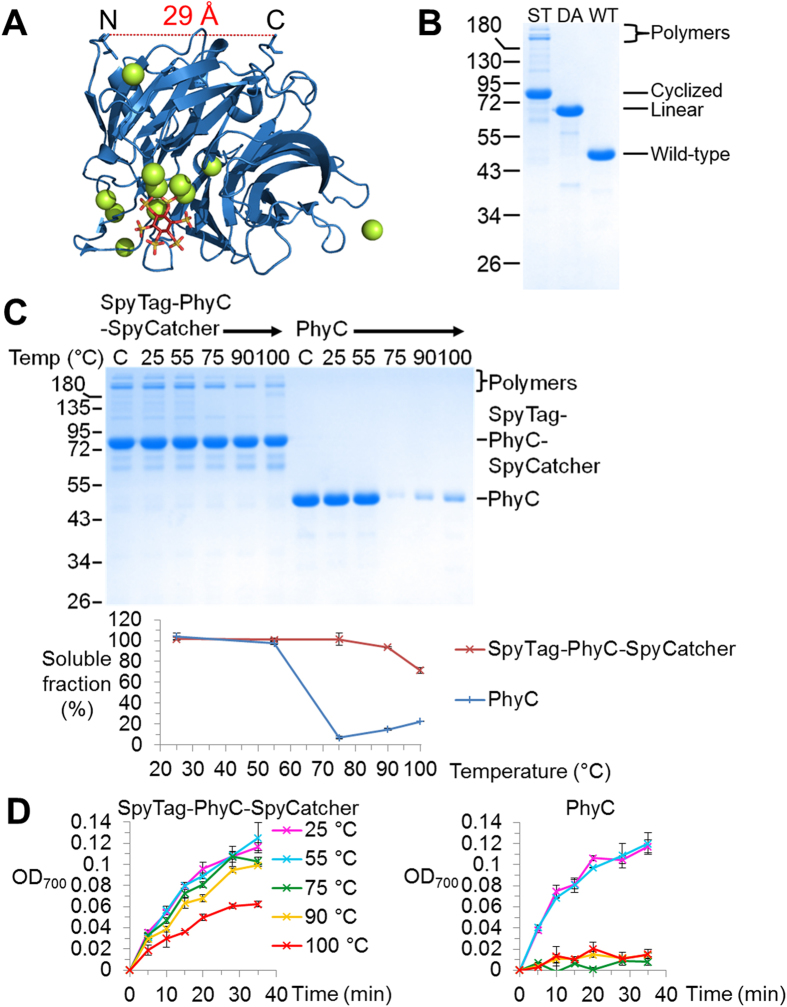
SpyRing cyclization of phytase increased the thermal resilience. (**A**) Crystal structure of PhyC phytase from *Bacillus subtilis* in cartoon format, with the substrate analog myo-inositol hexasulfate in stick format (based on PDB 3AMR). Spheres represent calcium ions and the distance between termini is marked. (**B**) Cyclization caused a gel shift. SpyTag-PhyC-SpyCatcher (ST), SpyTag DA-PhyC-SpyCatcher (DA) and PhyC (WT) were boiled in SDS-loading buffer and analyzed by SDS-PAGE with Coomassie staining. (**C**) SpyRing cyclization improved aggregation-resistance. SpyTag-PhyC-SpyCatcher or PhyC were heated at the indicated temperature for 10 min, centrifuged and the supernatant analyzed by SDS-PAGE with Coomassie staining. C is control without incubation. Data from triplicate measurements were then plotted. (**D**) SpyRing cyclization improved the recovered activity after heating. SpyTag-PhyC-SpyCatcher or PhyC were incubated for 10 min at the indicated temperature, before returning to RT and then running a colorimetric activity assay for phosphate release from phytic acid. (All are mean of triplicate ± 1 s.d.; some error bars are too small to be visible.)

We previously showed how SpyRing cyclization conferred resilience on BLA (7 Å between termini) and dihydrofolate reductase (15 Å between termini)^10^. To explore an industrially-important enzymatic activity with a much larger distance between termini, we tested cyclization of a phytase. The crystal structure of PhyC phytase from *Bacillus subtilis* has been previously determined^24^: the direct distance between the resolved N- and C-termini of PhyC is 29 Å (Fig. 4A). SpyTag-PhyC-SpyCatcher cyclized efficiently, based on the loss of H_2_O from isopeptide bond formation in MS (Fig. S4A) and the reduction in gel mobility compared to the non-cyclized SpyTag DA-PhyC-SpyCatcher control (Fig. 4B). SpyTag-PhyC-SpyCatcher remained active, exhibiting comparable specific activity to PhyC (Fig. S5). Heat-inactivation of phytases is a major challenge limiting their application for improving nutrition^18^. PhyC remained soluble after heating at 55 °C but aggregated at 75 °C (Fig. 4C). SpyRing cyclization led to a major increase in PhyC thermal resilience, with most of the enzyme remaining in solution even after heating at 100 °C (Fig. 4C). Most of PhyC’s catalytic activity was lost following 75 °C heating, but the SpyRing PhyC retained most of its activity even after 90 °C heating (Fig. 4D).

The non-cyclized control, SpyTag DA-PhyC-SpyCatcher behaved similarly to PhyC in both aggregation-resistance and recovery of catalytic activity following heating, so covalent cyclization was essential for SpyRing-mediated resilience of PhyC (Fig. S4).

### Secondary structure and calorimetric investigation of SpyRing phytase resilience

**Figure 5:**
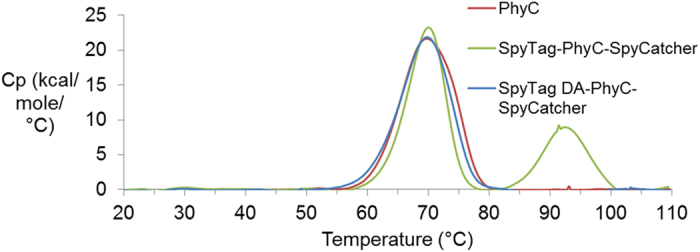
Analysis of SpyRing phytase by DSC. DSC of SpyTag (DA)-PhyC-SpyCatcher and PhyC. DSC gave the specific heat capacity (Cp) of each enzyme, scanning from 20–110 °C at 1 °C/min.

To understand better the basis for SpyRing phytase resilience, we looked for signatures of unfolding transitions upon heating. The Tm for unfolding of PhyC by DSC was not substantially shifted upon cyclization (PhyC 69.8 °C, SpyTag-PhyC-SpyCatcher 70.0 °C) (Fig. 5). For SpyTag-PhyC-SpyCatcher (but not PhyC or SpyTag DA-PhyC-SpyCatcher) a 2^nd^ transition was seen at 91.5 °C, which is likely to correspond to unfolding of the SpyTag/SpyCatcher domain (Fig. 5). This temperature is slightly higher than the 85.0 °C SpyTag/SpyCatcher transition from SpyTag-BLA-SpyCatcher (Fig. 3A). The difference in Tm might be due to a difference in buffer used for DSC (Tm of 85.0 °C in PBS pH 7.4 and Tm of 91.5 °C in 50 mM Tris-HCl pH 7.0 with 2 mM CaCl_2_) or might be an effect of PhyC stabilizing the SpyTag/SpyCatcher domain.

We then used circular dichroism (CD) to explore how heating affected secondary structure. We measured the ellipticity of the sample from 185–260 nm. For PhyC and SpyTag DA-PhyC-SpyCatcher there was little change in the spectrum at 55 °C, but at 75 °C and higher there was a general loss of signal, which did not recover upon cooling (Fig. S6). We observed aggregation for these proteins in the cuvette. However, for SpyRing phytase there was less perturbation at 75 and 90 °C (Fig. S6). The CD signal for SpyRing phytase did recover after cooling from 75 and 90 °C (Fig. S6), although interestingly the recovery was less complete than anticipated from the recovery of catalytic activity (Fig. 4D).

### SpyRing enzymes could be purified simply by heating

**Figure 6:**
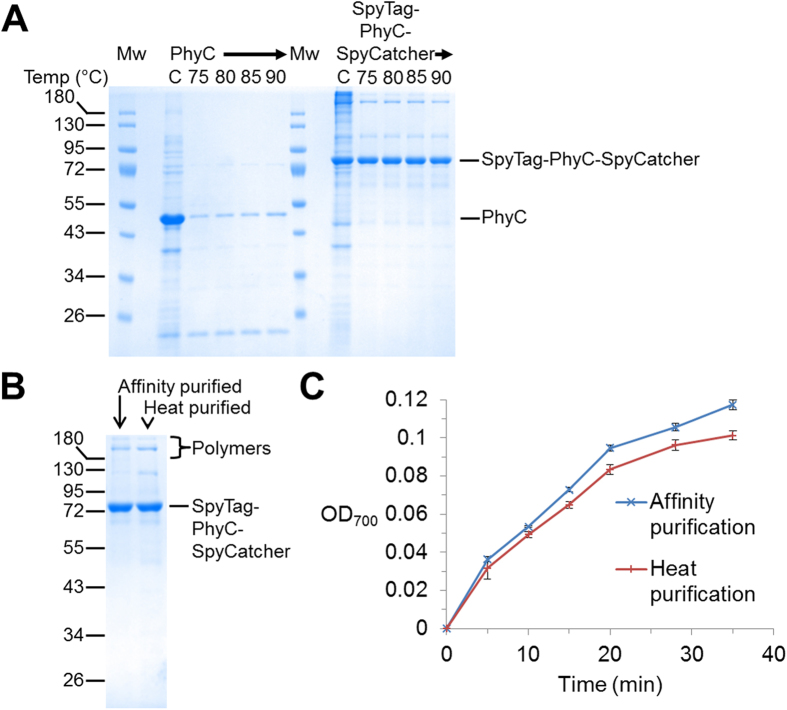
SpyRing cyclization allowed enzyme purification just by heating. (**A**) Heat-based purification of phytase. *E. coli* lysate expressing PhyC or SpyTag-PhyC-SpyCatcher was heated for 10 min at the indicated temperature, centrifuged, and the supernatant analyzed by SDS-PAGE with Coomassie staining. C is control without incubation. Mw are the molecular weight markers. (**B**) Comparing purity between heat-purified and affinity-purified phytase. SpyTag-PhyC-SpyCatcher either affinity-purified by Ni-NTA or heat-purified (normalized to give an equal intensity of the SpyTag-PhyC-SpyCatcher band) was boiled in SDS-loading buffer and analyzed by SDS-PAGE with Coomassie staining. (**C**) Heat purified phytase was still active. Catalytic activity of SpyTag-PhyC-SpyCatcher purified by either affinity chromatography or by heat purification (normalized by densitometry for an equal intensity of the SpyTag-PhyC-SpyCatcher band) was tested by a colorimetric activity assay for phosphate release from phytic acid. (All are mean of triplicate ± 1 s.d.; some error bars are too small to be visible.)

Enzyme purification often uses multiple chromatography steps, which can be time-consuming and expensive. We hypothesized that the ability of SpyRing cyclization to give extreme resilience to heating could permit a simple route to purification, since most of the endogenous cellular proteins might aggregate with heating^25^. Rather than starting with purified enzymes as previously, we explored heating the lysate obtained from enzyme over-expression in *E. coli*. PhyC was the major band on the Coomassie-stained gel of *E. coli* total cell lysate. While the heating at 75–90 °C removed most of the background proteins, heating also aggregated most of the PhyC (Fig. 6A). However, the SpyTag-PhyC-SpyCatcher band in the lysate showed minimal change in intensity after heating from 75–90 °C, while most endogenous proteins were depleted (Fig. 6A). Since 75 °C heating gave similar depletion of endogenous proteins as 90 °C (Fig. 6A), 75 °C heating was used subsequently. Typically the first step in purification does not purify to homogeneity, but SpyTag-PhyC-SpyCatcher heat purification gave a good purity in comparison to Ni-NTA purification (Fig. 6B). There were two major impurities from heat purification of SpyTag-PhyC-SpyCatcher at ~110 kDa and >180 kDa, neither of which were present in the heated PhyC sample (Fig. 6B). Therefore we hypothesize that the >180 kDa band is a product of intermolecular SpyTag-PhyC-SpyCatcher reaction.

Performing a similar approach for heat purification of SpyRing BLA also showed good recovery, with some impurities present and also more recovery of heat-stable SpyTag-BLA-SpyCatcher polymers than with Ni-NTA purification (Fig. S7A,B). Importantly for both SpyRing phytase (Fig. 6C) and SpyRing BLA (Fig. S7C), the recovered protein had comparable catalytic activity from heat purification when compared to affinity purification.

## Discussion

We have shown that cyclization with SpyTag/SpyCatcher gave superior resilience to other spontaneous isopeptide-forming domains of distinct sizes and reactive chemistries. SpyRing cyclization conferred resilience on phytase, an industrially-important class of enzyme with termini 29 Å apart. The mechanism of SpyRing resilience was studied through DSC and CD, indicating that cyclization by an isopeptide bond-forming domain did not change the temperature for initial unfolding but is likely to facilitate refolding. The resilience to thermal inactivation of SpyRings allowed normally heat-labile enzymes to be purified simply by heating of cell lysate.

The alternative isopeptide-forming partners, Pilin-C/Isopeptag and SnoopCatcher/SnoopTag, allowed efficient enzyme cyclization, even though their reaction rate (when tested intermolecularly) is slower than SpyCatcher/SpyTag^11^,^19^,^21^. Both PilinRing and SnoopRing cyclization gave resilience to heat-induced aggregation/inactivation, even at 100 °C, so enzyme resilience from cyclization by isopeptide bond-forming domains is a general phenomenon. Non-reactive point mutants confirmed that isopeptide bond formation was important to the effect on enzyme resilience. Inability to form the isopeptide bond had two effects: lowering the Tm of the fused partner and untethering the termini, both likely to decrease the enzyme’s resilience. The degree of resilience from PilinRing or SnoopRing cyclization for BLA was not as complete as with SpyRing cyclization. For PilinRing and SnoopRing cyclization, recovery of activity after 75 °C heating was less efficient than after 100 °C heating. Previous studies have suggested that temperature may influence the partition between different unfolded states, some of which favor refolding and some of which favor aggregation^26^,^27^. The importance of the cyclization framework for determining the extent of resilience is consistent with the large difference in BLA thermo-resilience reported through split intein cyclization (~5 °C increase)^5^ compared to SpyRing cyclization (>60 °C increase)^10^. It is also consistent with our previous work which showed that the cyclized SpyTag-BLA-SpyCatcher construct exhibited a greater thermal resilience than a BLA-CnaB2 fusion construct^10^. PilinRing cyclization did give excellent resistance to aggregation, but after heating not all the soluble enzyme was functional. Resilience did not correlate with the size of the cyclizing domain, since Pilin-C is much bigger than SpyCatcher, but resilience did correlate with the unfolding temperature of the cyclizing domain. Interestingly all three cyclization systems stabilized the sandwiched enzyme from aggregation at 100 °C where DSC indicates that the Tag/Catcher domain would have unfolded.

SpyRing cyclization had a major effect in increasing the thermal resilience of PhyC phytase. Extensive efforts have been made to engineer different classes of phytases for high stability but phytase inactivation when food pellets are heat-sterilized is still a significant issue and so our SpyRing phytase provides a new direction for this challenge^18^,^28^. Phytase supplementation has already shown success in human trials for improving dietary uptake of iron and zinc^29^. Alternatives to phytase supplementation include the engineering of crops expressing phytase^18^ or “Enviropig”, a genetically-modified pig producing phytase in its saliva^30^. However, eating transgenic organisms encounters a lot of resistance from the general public^31^.

It is commonly known that enzymes from hyperthermophiles, such as Taq DNA polymerase from *Thermus aquaticus*, can be expressed in *E. coli* and purified simply by heating^25^,^32^. Establishing such a simple purification route for enzymes that are not intrinsically thermostable could have academic and industrial importance. We showed here that SpyRing cyclization allowed substantial heat-purification of BLA and phytase with high retention of activity. Purification was not dissimilar to that achieved by affinity purification, although the protein was not purified to homogeneity^33^. Nevertheless orthogonal polishing steps may then be used to purify further. Also, for many industrial processes, such as nutritional supplementation or biotransformations, moderate purity is often sufficient. Fusing proteins to thermostable domains was previously shown to allow stabilization^34^ and heat-purification of proteins of interest, but this required the protein of interest to be substantially smaller than the thermostable domain^35^. In contrast, here SpyRing cyclization was effective in conferring resilience on fused enzymes substantially larger than SpyTag/SpyCatcher.

SpyRings represent a simple and versatile approach to increase the thermal resilience of enzymes. Future work will apply this technology to other globally-important enzymes and endeavor to improve resilience even further, by increasing SpyTag/SpyCatcher’s Tm or by harnessing the bacterial diversity of isopeptide-forming domains^36^.

## Methods

### Cloning

KOD Hot Start DNA Polymerase (Roche) was used as the primary polymerase for PCR. DNA was transformed into competent *E. coli* DH5α (Life Technologies). All constructs contained an N-terminal His_6_ tag encoded in the pET28a plasmid (Novagen). The *Bacillus subtilis* PhyC insert (GenBank: JQ437256.1) was assembled using the DNAWorks method^37^. The PhyC insert was amplified using primers 5′- CCGACGAAGGGTTCAGGGGGTTCCGGTAAACATAAACTGAGCGATCCGTATCATTTCAC and 5′- GCGCCTCCGCTGCCACCACTCCCTTTGCCCGAACGATCGGTCAATTTACG. The insert was cloned into the SpyTag/SpyCatcher cassette using the method previously described^10^ to make pET28a SpyTag-PhyC-SpyCatcher (GenBank accession number KU608330) and pET28a SpyTag DA-PhyC-SpyCatcher. pET28a PhyC without the SpyTag/SpyCatcher fusions was cloned by amplifying the PhyC gene from pET28a SpyTag-PhyC-SpyCatcher using primers 5′- TATACATATGGGAAAACATAAACTGAGCGATCCGTATCATTTCAC and 5′- TTTTAAGCTTTCATTATTTGCCCGAACGATCGGTCAATTTACG. The amplified product was inserted into pET28a using restriction digestion with NdeI (NEB) and HindIII (NEB), followed by ligation using T4 DNA ligase (NEB). pET28a SpyTag-BLA-SpyCatcher (GenBank KJ645919, Addgene ID 70943), pET28a SpyTag DA-BLA-SpyCatcher and pET28a BLA were described previously^10^. pDEST14 SpyCatcher^11^, pET28a Spy0128^19^ and pET28a RrgACatcher G842T (SnoopCatcher G848D)^21^ were as described. pET28a SnoopTag-BLA-SnoopCatcher (GenBank accession number KU608331) was cloned using overlap extension PCR^38^ followed by digestion with NdeI and HindIII and ligation into pET28a. SnoopTag is based on the N-terminal β-strand of RrgA’s D4 domain (residues 734–748, numbering from PDB ID 2WW8)^22^. SnoopCatcher is based on residues 749–860 of *Streptococcus pneumoniae* adhesin RrgA’s D4 domain (numbering from PDB ID 2WW8). The first fragment containing the SnoopTag-BLA component was amplified from SpyTag-BLA-SpyCatcher using primers 5′- ATTACATATGGGAAAACTGGGGGACATCGAATTCATCAAAGTAAACAAAGGTTCAGGGGGTTCCGGTCACC and 5′- CTAAACACGGCACCACGCAGCGGCTTTCCACTGCCACCACTCCCCCAATGC. The second fragment containing the SnoopCatcher component was amplified from the RrgACatcher G842T construct^21^ using primers 5′- AAGCCGCTGCGTGGTGCCG and 5′- ATTAAAGCTTTCATTATTTCGGCGGTATCGGTTCATTGGTG. pET28a SnoopTag KA-BLA-SnoopCatcher (K742 to A) was generated using pET28a SnoopTag-BLA-SnoopCatcher as a template and primer 5′- CCCTGAACCTTTGTTTACTGCGATGAATTCGATGTCCCC and its reverse complement by QuikChange. pET28a Pilin-C-BLA-Isopeptag (GenBank accession number KU608332) was cloned using Gibson assembly^39^ with Gibson Assembly Master Mix (NEB). The vector was amplified using primers 5′- TCCCATATGGCTGCCGCGCGG and 5′- AAGCTTGCGGCCGCACTCGAGC using pET28a SpyTag-BLA-SpyCatcher as a template. The Pilin-C fragment was generated using primers 5′- CCGCGCGGCAGCCATATGGGAGCTACAACAGTTCACGGGGAGAC and 5′- GGTGACCGGAACCCCCTGAACCAATGGTCATATCTTTATCAGTAGATGTCTCTTG using pET28a Pilin-C^19^ as a template. The BLA-Isopeptag fragment was amplified using primers 5′- GGTTCAGGGGGTTCCGGTCAC and 5′- GCTCGAGTGCGGCCGCAAGCTTTCATTATTCCGCATCTTTTTTGTTGGTAAAGGTAATGGTCATATCTTTATCGGTTCCACTGCCACCACTCCCCC using pET28a SpyTag-BLA-SpyCatcher as a template. pET28a Pilin-C KA-BLA-Isopeptag was generated using pET28a Pilin-C-BLA-Isopeptag as a template and primer 5′- TCTACTACATTAACGGTGAAGGCAAAAGTTTCAGGTA CCGGTGG and its reverse complement by QuikChange. All constructs were verified by Sanger sequencing.

### Recombinant protein expression and purification

Samples for MS were expressed in *E. coli* B834 DE3 cells (Novagen) in the absence of glucose to avoid gluconylation^40^. Saturated cultures were diluted 1 in 100 and grown at 37 °C in LB with 0.5 mg/mL kanamycin to an OD_600_ of 0.5 and induced using 0.4 mM isopropyl β-D-1-thiogalactopyranoside (IPTG, Melford Labs) at 30 °C for 3 hours. For all other purposes, samples were expressed in *E. coli* BL21 DE3 RIPL (Stratagene), except Pilin-C (KA)-BLA-Isopeptag was expressed in *E. coli* B834 DE3. Samples were grown in LB with 0.8% glucose and 0.5 mg/mL kanamycin overnight. Saturated cultures were diluted 1 in 100 and grown at 37 °C until cultures reached an OD_600_ of 0.5. The cultures were induced with 0.4 mM IPTG and grown for 16 hours at 18 °C. Samples were spun down at 4,000 g for 20 minutes and the pellet was re-suspended in 50 mM Tris-HCl pH 7.8 with 300 mM NaCl (Ni-NTA buffer) containing 1 mM phenylmethylsulfonyl fluoride (PMSF, Roche) and 1× EDTA-free mixed protease inhibitors (Roche). Protein purification was performed using Ni-NTA resin (Qiagen). Samples were allowed to bind to the resin for 30 minutes at 4 °C. The sample was added onto a poly-prep column (Bio-Rad), washed with 40 resin volumes of Ni-NTA buffer with 10 mM imidazole, then washed with 20 resin volumes of Ni-NTA buffer with 30 mM imidazole and eluted with 4 resin volumes of Ni-NTA buffer with 75 mM imidazole. SpyTag (DA)-BLA-SpyCatcher, BLA, SnoopTag (KA)-BLA-SnoopCatcher, and Pilin-C (KA)-BLA-Isopeptag samples used in solubility assays, enzymatic assays and SDS-PAGE assays were dialyzed 3 times in PBS. SpyTag (DA)-PhyC-SpyCatcher and PhyC samples used in solubility assays, enzymatic assays and SDS-PAGE assays were dialyzed 3 times in 50 mM Tris-HCl pH 7.0 with 2 mM CaCl_2_. SnoopTag (KA)-BLA-SnoopCatcher, Pilin-C (KA)-BLA-Isopeptag, and SpyTag (DA)-PhyC-SpyCatcher samples for MS were dialyzed 3 times in 10 mM ammonium acetate. SpyTag (DA)-PhyC-SpyCatcher and PhyC samples used for CD were dialyzed 3 times into 10 mM Tris-HCl pH 8.0 with 2 mM CaCl_2_ and then purified again using anion-exchange to polish the samples and remove polymeric species. Anion-exchange chromatography was performed using a 1 mL HiTrap HP Q column (GE Healthcare) on an ÄKTA purifier (GE Healthcare). Fractions were collected and concentrated using a Vivaspin 6 centrifugal concentrator (Sartorius) with a 5 kDa cut-off and dialyzed 3 times in 10 mM Tris-SO_4_ (Tris base with pH adjusted using H_2_SO_4_) pH 7.0 with 2 mM CaSO_4_. Samples used to obtain values for kinetic parameters or units of activity were further purified by gel filtration chromatography to remove multimers. Gel filtration was performed using a Superdex 200 10/300 GL column (GE Healthcare) on an ÄKTA purifier. Fractions containing only enzyme monomer were collected and concentrated using a Vivaspin 6 centrifugal concentrator with a 5 kDa cut-off. Concentrations were determined from OD_280_ and the calculated molar extinction coefficients from ProtParam. For the comparative kinetic and activity studies, protein concentrations were determined using a Pierce bicinchoninic acid (BCA) Protein Assay Kit (Thermo Fisher Scientific).

### Reaction of SpyCatcher with SpyTag peptide and SnoopCatcher with SnoopTag peptide

SpyTag peptide (GAHIVMVDAYKPTK) and SnoopTag peptide (GKLGDIEFIKVNKGY) were synthesized by Insight Biotechnology at >95% purity, validated by HPLC and mass spectrometry. SpyCatcher was allowed to react with a 4-fold molar excess of SpyTag at 20 °C for 2 hours in PBS. SnoopCatcher was allowed to react with a 3-fold molar excess of SnoopTag at 20 °C for 20 hours in PBS. Each sample was then dialyzed 3 times in PBS. The reaction was confirmed by the gel shift on 20% SDS-PAGE^19^.

### SDS-PAGE

SDS-PAGE was run on 12% (for phytase constructs and heat purification gels) or 14% (for β-lactamase constructs) polyacrylamide gels in an XCell SureLock module (Life Technologies). Gels were run at 200 V for 1 hour and then stained with InstantBlue Coomassie (Expedeon). For β-lactamase constructs, a final concentration of 100 mM dithiothreitol (DTT, Sigma) was added. For phytase constructs, a final concentration of 100 mM ethylenediaminetetraacetic acid (EDTA) was added to remove tightly-bound divalent cations affecting phytase mobility. Samples were mixed with 6× SDS-PAGE loading buffer (0.23 M Tris-HCl, 0.24% glycerol, 6.7% SDS, and 12 mM bromophenol blue) and heated at 95 °C for 7 minutes in a Bio-Rad C1000 Thermal cycler. Gels were imaged on a Bio-Rad ChemiDoc XRS+ and analyzed using Image Lab 3.0 software (Bio-Rad).

### Temperature-dependent solubility assay

20 μL 25 μM BLA construct in PBS with 100 mM DTT was incubated at 10, 25, 37, 55, 75, 90 or 100 °C for 10 minutes and then cooled to 10 °C on a Bio-Rad C1000 Thermal Cycler. 20 μL 10 μM phytase construct in 50 mM Tris-HCl pH 7.0 with 2 mM CaCl_2_ was incubated at 10, 25, 55, 75, 90 or 100 °C for 10 minutes and then cooled to 10 °C on a Bio-Rad C1000 Thermal Cycler. The ramp rate was 3 °C/s. Samples were spun at 17,000 g at 4 °C for 30 minutes and the supernatant was removed for SDS-PAGE. Every gel had a triplicate loading control to normalize the samples. Loading samples consisted of the protein sample that had been kept at 10 °C. The loading control was defined as 100% soluble fraction. Bands were quantified using densitometry values obtained from the Image Lab 3.0 software.

### β-lactamase thermal resilience enzymatic assay

A 96 well clear flat-bottom polystyrene plate (Greiner) was pre-blocked with 200 μL PBS with 3% bovine serum albumin (BSA, ≥ 98%, Sigma) at 37 °C for 2 hours. The plate was then rinsed twice using 0.1 M NaH_2_PO_4_ pH 7.0 with 1 mM EDTA. 20 μL 25 μM BLA construct in PBS with 100 mM DTT was incubated at 10, 25, 37, 55, 75, 90 or 100 °C for 10 minutes and then cooled to 10 °C on a Bio-Rad C1000 Thermal Cycler (3 °C/s). The protein was diluted 1 in 400 using 0.1 M NaH_2_PO_4_ pH 7.0 with 1 mM EDTA and 1% BSA. 3 nM of BLA construct was allowed to react with 100 μM nitrocefin (Merck) in 0.1 M NaH_2_PO_4_ pH 7.0 with 1 mM EDTA at 20 °C. The reaction was quenched at different time-points by adding an equal volume of 500 μM potassium clavulanate (Sigma) dissolved in 0.1 M NaH_2_PO_4_ pH 7.0 with 1 mM EDTA. Care was taken to protect the plates from direct light. Half of the volume was transferred into a clean 96 well clear flat-bottom polystyrene plate to remove bubbles that could interfere with absorbance measurements. OD_486_ was measured using a SpectraMax M3 microplate reader (Molecular Devices). Absorbance was corrected for the dilution caused by the addition of potassium clavulanate. Samples were blanked against a control lacking enzyme.

### β-lactamase kinetics assay

BLA activity was measured as previously^41^. 2.0 mL sample cups (Kartell Labware) were pre-blocked using 50 mM NaH_2_PO_4_ pH 7.0 with 3% BSA at 37 °C for 2 hours. The samples cups were then rinsed twice using 50 mM NaH_2_PO_4_ pH 7.0. Enzymes were diluted in the sample cup to a concentration of 0.25 nM using 50 mM NaH_2_PO_4_ pH 7.0 with 0.1% BSA. Samples were then allowed to react in 50 mM NaH_2_PO_4_ pH 7.0 with 100, 50, 30, 20, 15, or 10 μM Nitrocefin (Oxoid) in a Gallery Plus Automated Photometric Analyzer (Thermo Scientific) at 30 °C. Readings were taken every 9 seconds for a period of 99 seconds at a wavelength of 500 nm (Δε_500_ = 15,900 M^−1^cm^−1^)^42^. The first 6 data points were linear and were used to obtain the initial rates of reaction. A Lineweaver-Burk plot was used to extrapolate the kinetic parameters. Errors for kinetic values were obtained by calculating the kinetics for 3 separate sets of data and then calculating the average and standard deviation of the 3 kinetic values.

### Phytase thermal resilience enzymatic assay

A 96 well clear flat-bottom polystyrene plate (Greiner) was pre-blocked with 200 μL 50 mM Tris-HCl pH 7.0 with 2 mM CaCl_2_ and 3% BSA at 37 °C for 2 hours. The plate was then rinsed twice using 50 mM Tris-HCl pH 7.0 with 2 mM CaCl_2_. 20 μl of 10 μM phytase construct in 50 mM Tris-HCl pH 7.0 with 2 mM CaCl_2_ was incubated at 10, 55, 75, 90 or 100 °C for 10 minutes and then cooled to 10 °C on a Bio-Rad C1000 Thermal Cycler (3 °C/s). The protein was diluted 1 in 26 using 50 mM Tris-HCl pH 7.0, 2 mM CaCl_2_, 10% glycerol, 1% BSA. 77 nM phytase construct was allowed to react with 2 mM phytic acid (Sigma) in 50 mM Tris-HCl pH 7.0 with 2 mM CaCl_2_ and 10% glycerol at 50 °C. The reaction was quenched at different time points by adding an equal volume of 20% trichloroacetic acid. A solution containing 12 mM ammonium heptamolybdate (Sigma) and 4.3% H_2_SO_4_ was mixed in a 4 : 1 ratio with a solution containing 178 mM FeSO_4_ (Scientific Laboratory Supplies) to make the color mix solution. 1 volume of color mix solution was added for every 2 volumes of quenched reaction solution. The plates were spun at 3,500 g at 20 °C in a Multifuge X3R (Thermo Scientific) for 10 minutes. Within 8 minutes, 60% of the solution (90 μL of 150 μL) was transferred into a clean 96 well clear flat-bottom polystyrene plate and OD_700_ was measured using a SpectraMax M3 microplate reader. Blank consisted of all the components minus phytase.

### Phytase specific activity assay

Phytase activity was measured as previously^43^. 40 nM phytase was incubated with 1.6 mM phytic acid at 37 °C for 1, 2, 3, 4, or 5 minutes in 100 mM Tris-HCl pH 7.5 with 1 mM CaCl_2_. The reaction was quenched by adding an equal volume of 20% trichloroacetic acid. Samples were spun at 17,000 g at 20 °C for 10 minutes and 90% of the supernatant was moved into a clean tube. A solution containing 12 mM ammonium heptamolybdate (Sigma) and 4.3% H_2_SO_4_ was mixed in a 4:1 ratio with a solution containing 178 mM FeSO_4_ (Scientific Laboratory Supplies) to make the color mix solution. 1 volume of color mix solution was added for every 2 volumes of quenched reaction solution. The samples were then incubated at 25 °C for 20 minutes and then OD_700_ was measured using a Varian Cary 50 UV-Vis Spectrophotometer. For the blank, the enzyme was first added to 20% trichloroacetic acid to inactivate the enzyme before mixing with the substrate. In order to determine the specific activity, OD_700_ was converted to μg of phosphate using a set of standards containing 20 to 100 μM NaH_2_PO_4_. A straight line of best fit was used to obtain the initial rate. The specific activity for each repeat was individually calculated and averaged. The error refers to 1 standard deviation from the mean.

### Heat purification

Bacterial pellets obtained from an 800 mL bacterial culture were frozen at −80 °C and then thawed. Cells were resuspended in 8 mL 50 mM Tris-HCl pH 7.8 with 300 mM NaCl containing 1 mM phenylmethylsulfonyl fluoride (PMSF, Roche) and 1× EDTA-free mixed protease inhibitors (Roche, 1 tablet resuspended in 1.5 ml H_2_O was 100× stock). The OD_600_ of the solution was 110–150 (as determined on a diluted sample of the resuspended cells). Cells were sonicated and centrifuged at 17,000 g for 20 minutes at 4 °C. The soluble fraction was removed. The OD_280_ of the solution was 60 (as determined on a diluted sample). Half of the cell lysate was purified using Ni-NTA affinity chromatography as described above and dialyzed 3 times into 50 mM Tris-HCl pH 7.0 with 2 mM CaCl_2_ (PhyC constructs) or PBS pH 7.4 (BLA constructs) for use in the activity comparison later on. The other half was diluted 1 in 10 into 50 mM Tris-HCl pH 7.0 with 2 mM CaCl_2_ (PhyC constructs) or PBS pH 7.4 (BLA constructs) The sample was split into 50 μL aliquots and heated at 75, 80, 85 or 90 °C for 10 minutes in a Bio-Rad C1000 Thermal Cycler and then cooled to 10 °C. Samples were then centrifuged at 17,000 g at 4 °C for 30 minutes and the supernatant was removed and further analyzed. Samples heated to 75, 80, 85 or 90 °C were analyzed via SDS-PAGE to check their purity. For testing purity and activity, the concentration of SpyTag-PhyC-SpyCatcher from affinity purification was matched to the concentration of SpyTag-PhyC-SpyCatcher from heat purification using densitometry of the SpyTag-PhyC-SpyCatcher band based on SDS-PAGE with Coomassie staining.

### Mass spectrometry

The samples were analyzed using a Micromass LCT time-of-flight electrospray ionization mass spectrometer (Micromass UK). MassLynx V 4.00.00 (Waters Corporation) was used to convert the m/z spectrum to a mass spectrum using a Maximum Entropy algorithm. Predicted masses were obtained using ProtParam and by taking into account the change in mass that occurs when an isopeptide bond is formed (−18 for SpyCatcher, −17 for SnoopCatcher and Pilin-C)^10^,^20^,^22^.

### DSC

DSC profiles of PhyC, SpyTag (DA)-PhyC-SpyCatcher, SpyTag-BLA-SpyCatcher, SnoopTag (KA)-BLA-SnoopCatcher, Pilin-C (KA)-BLA-Isopeptag, BLA, Spy0128, SpyCatcher + SpyTag and SnoopCatcher + SnoopTag were measured on the VP Cap DSC (GE Healthcare). The DSC conditions to determine the Tm were 20 μM protein in PBS pH 7.4 (BLA constructs, Spy0128, SpyCatcher + SpyTag and SnoopCatcher +SnoopTag) or 50 mM Tris-HCl pH 7.0 with 2 mM CaCl_2_ (PhyC constructs) from 20 to 110 °C at a scan rate of 1 °C/min at a pressure of 3 atm. The baseline traces were subtracted from the sample traces and then normalized for concentration and volume using the MicroCal DSC Origin Pro 7.0 software (GE Healthcare). Samples containing only buffer were used to obtain the baseline traces.

### CD

Circular Dichroism experiments were performed in 10 mM Tris-SO_4_ pH 7.0 with 2 mM CaSO_4_ buffer. Samples were analyzed on a J-815 Circular Dichroism Spectrometer (Jasco) using a 1 mm path-length Spectrosil quartz cuvette (Starna Scientific). The temperature was controlled using a CDF-426S Peltier unit (Jasco). Far-UV CD spectra were obtained by incubating 0.15 mg/mL protein at 10 °C, before ramping at 12 °C/min to 55, 75 or 90 °C. Samples were held at that temperature for 3 minutes before starting a read (the “During heating” trace). The scanning speed of the instrument was set to 50 nm/min and scans were performed from 260 to 185 nm. After the read, samples were cooled to 10 °C. Samples were kept at 10 °C for 3 minutes before taking another read (the “After cooling” trace). Four scans were recorded from each independent sample, the recorded spectra were averaged, and then subtracted by the averaged baseline spectrum (buffer alone). Spectra were smoothened using the Savitzky-Golay method^44^ on the SpectraManager 2 software (Jasco). Ellipticity was converted to molar ellipticity using the SpectraManager 2 software.

### Molecular visualization

Protein structures were rendered in PyMOL (DeLano Scientific), based on Protein Data Bank files 1ZG4^45^, 3B2M^20^, 2WW8^22^, and 3AMR^24^.

## Additional Information

**How to cite this article**: Schoene, C. *et al*. SpyRing interrogation: analyzing how enzyme resilience can be achieved with phytase and distinct cyclization chemistries. *Sci. Rep.* **6**, 21151; doi: 10.1038/srep21151 (2016).

## Supplementary Material

Supplementary Information

## References

[b1] Kawedia J. D. & Rytting M. E. Asparaginase in Acute Lymphoblastic Leukemia. Clin. Lymphoma Myeloma Leuk. 14, Supplement, S14–S17 (2014). 25486949 10.1016/j.clml.2014.06.017

[b2] Bhosale S. H., Rao M. B. & Deshpande V. V. Molecular and industrial aspects of glucose isomerase. Microbiol. Rev. 60, 280–300 (1996). 8801434 10.1128/mr.60.2.280-300.1996 PMC239444

[b3] Reetz M. T. & Carballeira J. D. Iterative saturation mutagenesis (ISM) for rapid directed evolution of functional enzymes. Nat. Protoc. 2, 891–903 (2007). 17446890 10.1038/nprot.2007.72

[b4] Evans T. C. Jr, Benner J. & Xu M. Q. The *in vitro* ligation of bacterially expressed proteins using an intein from Methanobacterium thermoautotrophicum. J. Biol. Chem. 274, 3923–3926 (1999). 9933578 10.1074/jbc.274.7.3923

[b5] Iwai H. & Plückthun A. Circular β-lactamase: stability enhancement by cyclizing the backbone. FEBS Lett. 459, 166–172 (1999). 10518012 10.1016/s0014-5793(99)01220-x

[b6] Camarero J. A. & Muir T. W. Biosynthesis of a Head-to-Tail Cyclized Protein with Improved Biological Activity. J. Am. Chem. Soc. 121, 5597–5598 (1999).

[b7] Scott C. P., Abel-Santos E., Wall M., Wahnon D. C. & Benkovic S. J. Production of cyclic peptides and proteins *in vivo*. Proc. Natl. Acad. Sci. 96, 13638–13643 (1999). 10570125 10.1073/pnas.96.24.13638 PMC24117

[b8] Parthasarathy R., Subramanian S. & Boder E. T. Sortase A as a novel molecular ‘stapler’ for sequence-specific protein conjugation. Bioconjug. Chem. 18, 469–476 (2007). 17302384 10.1021/bc060339w

[b9] Goldenberg D. P. & Creighton T. E. Circular and circularly permuted forms of bovine pancreatic trypsin inhibitor. J. Mol. Biol. 165, 407–413 (1983). 6188846 10.1016/s0022-2836(83)80265-4

[b10] Schoene C., Fierer J. O., Bennett S. P. & Howarth M. SpyTag/SpyCatcher Cyclization Confers Resilience to Boiling on a Mesophilic Enzyme. Angew. Chem. Int. Ed. 53, 6101–6104 (2014). 10.1002/anie.201402519 PMC4286826 24817566

[b11] Zakeri B. . Peptide tag forming a rapid covalent bond to a protein, through engineering a bacterial adhesin. Proc. Natl. Acad. Sci. 109, E690–E697 (2012). 22366317 10.1073/pnas.1115485109 PMC3311370

[b12] Veggiani G., Zakeri B. & Howarth M. Superglue from bacteria: unbreakable bridges for protein nanotechnology. Trends Biotechnol. 32, 506–512 (2014). 25168413 10.1016/j.tibtech.2014.08.001 PMC4281928

[b13] Bailey R. L., West K. P. & Black R. E. The epidemiology of global micronutrient deficiencies. Ann. Nutr. Metab. 66 Suppl 2, 22–33 (2015). 26045325 10.1159/000371618

[b14] Allen L., de Benoist B., Dary O. & Hurrell R. Guidelines on Food Fortification with Micronutrients. (World Health Organization, 2006).

[b15] Ye X. . Engineering the Provitamin A (β-Carotene) Biosynthetic Pathway into (Carotenoid-Free) Rice Endosperm. Science 287, 303–305 (2000). 10634784 10.1126/science.287.5451.303

[b16] Bohlke R. A., Thaler R. C. & Stein H. H. Calcium, phosphorus, and amino acid digestibility in low-phytate corn, normal corn, and soybean meal by growing pigs. J. Anim. Sci. 83, 2396–2403 (2005). 16160052 10.2527/2005.83102396x

[b17] Selle P. H., Ravindran V., Bryden W. L. & Scott T. Influence of Dietary Phytate and Exogenous Phytase on Amino Acid Digestibility in Poultry: A Review. J. Poult. Sci. 43, 89–103 (2006).

[b18] Lei X. G., Weaver J. D., Mullaney E., Ullah A. H. & Azain M. J. Phytase, a New Life for an ‘Old’ Enzyme. Annu. Rev. Anim. Biosci. 1, 283–309 (2013). 25387021 10.1146/annurev-animal-031412-103717

[b19] Zakeri B. & Howarth M. Spontaneous Intermolecular Amide Bond Formation between Side Chains for Irreversible Peptide Targeting. J. Am. Chem. Soc. 132, 4526–4527 (2010). 20235501 10.1021/ja910795a

[b20] Kang H. J., Coulibaly F., Clow F., Proft T. & Baker E. N. Stabilizing isopeptide bonds revealed in gram-positive bacterial pilus structure. Science 318, 1625–1628 (2007). 18063798 10.1126/science.1145806

[b21] Veggiani G. . Programmable polyproteams built using twin peptide superglue. Proc. Natl. Acad. Sci. doi: 10.1073/pnas.1519214113 2016. PMC4747704 26787909

[b22] Izoré T. . Structural basis of host cell recognition by the pilus adhesin from Streptococcus pneumoniae. Structure 18, 106–115 (2010). 20152157 10.1016/j.str.2009.10.019

[b23] Hagan R. M. . NMR Spectroscopic and Theoretical Analysis of a Spontaneously Formed Lys-Asp Isopeptide Bond. Angew. Chem. Int. Ed. 49, 8421–8425 (2010). 10.1002/anie.201004340 PMC3315829 20878961

[b24] Zeng Y.-F. . Crystal Structures of Bacillus Alkaline Phytase in Complex with Divalent Metal ions and Inositol Hexasulfate. J. Mol. Biol. 409, 214–224 (2011). 21463636 10.1016/j.jmb.2011.03.063

[b25] Kirk N. & Cowan D. Optimising the recovery of recombinant thermostable proteins expressed in mesophilic hosts. J. Biotechnol. 42, 177–184 (1995). 7576536 10.1016/0168-1656(95)00078-5

[b26] Wang W. & Roberts C. J. Non-Arrhenius Protein Aggregation. AAPS J. 15, 840–851 (2013). 23615748 10.1208/s12248-013-9485-3 PMC3691426

[b27] Sakamoto R., Nishikori S. & Shiraki K. High temperature increases the refolding yield of reduced lysozyme: implication for the productive process for folding. Biotechnol. Prog. 20, 1128–1133 (2004). 15296439 10.1021/bp034385b

[b28] Ping S.-Z., Zhang W., Lin M., Yan Y.-L., Zhao Z.-L., Lu W. & Chen M. Inventors; Biotechnology Research Institute, Chinese Academy of Agricultural Sciences, assignee. Cyclized phytase with improved heat stability and proteinase resistance. People’s Republic of China patent CN101638642 A. 2010 Feb 3.

[b29] Troesch B., Jing H., Laillou A. & Fowler A. Absorption studies show that phytase from Aspergillus niger significantly increases iron and zinc bioavailability from phytate-rich foods. Food Nutr. Bull. 34, S90–101 (2013). 24050000 10.1177/15648265130342S111

[b30] Golovan S. P. . Pigs expressing salivary phytase produce low-phosphorus manure. Nat. Biotechnol. 19, 741–745 (2001). 11479566 10.1038/90788

[b31] Wu F. Explaining public resistance to genetically modified corn: an analysis of the distribution of benefits and risks. Risk Anal. Off. Publ. Soc. Risk Anal. 24, 715–726 (2004). 10.1111/j.0272-4332.2004.00470.x 15209940

[b32] Tanaka T., Kawano N. & Oshima T. Cloning of 3-Isopropylmalate Dehydrogenase Gene of an Extreme Thermophile and Partial Purification of the Gene Product. J. Biochem. (Tokyo) 89, 677–682 (1981). 7016850 10.1093/oxfordjournals.jbchem.a133245

[b33] Kwon S., Jung Y. & Lim D. Proteomic analysis of heat-stable proteins in Escherichia coli. BMB Rep. 41, 108–111 (2008). 18315945 10.5483/bmbrep.2008.41.2.108

[b34] Pierre B., Xiong T., Hayles L., Guntaka V. R. & Kim J. R. Stability of a guest protein depends on stability of a host protein in insertional fusion. Biotechnol. Bioeng. 108, 1011–1020 (2011). 21190177 10.1002/bit.23039

[b35] de Marco A., Casatta E., Savaresi S. & Geerlof A. Recombinant proteins fused to thermostable partners can be purified by heat incubation. J. Biotechnol. 107, 125–133 (2004). 14711496 10.1016/j.jbiotec.2003.10.008

[b36] Kang H. J. & Baker E. N. Intramolecular isopeptide bonds: protein crosslinks built for stress? Trends Biochem. Sci. 36, 229–237 (2011). 21055949 10.1016/j.tibs.2010.09.007

[b37] Hoover D. M. & Lubkowski J. DNAWorks: an automated method for designing oligonucleotides for PCR-based gene synthesis. Nucleic Acids Res. 30, e43 (2002). 12000848 10.1093/nar/30.10.e43 PMC115297

[b38] Bryksin A. V. & Matsumura I. Overlap extension PCR cloning: a simple and reliable way to create recombinant plasmids. BioTechniques 48, 463–465 (2010). 20569222 10.2144/000113418 PMC3121328

[b39] Gibson D. G. . Enzymatic assembly of DNA molecules up to several hundred kilobases. Nat. Methods 6, 343–345 (2009). 19363495 10.1038/nmeth.1318

[b40] Geoghegan K. F. . Spontaneous alpha-N-6-phosphogluconoylation of a ‘His tag’ in Escherichia coli: the cause of extra mass of 258 or 178 Da in fusion proteins. Anal. Biochem. 267, 169–184 (1999). 9918669 10.1006/abio.1998.2990

[b41] Raquet X. . TEM β-Lactamase Mutants Hydrolysing Third-generation Cephalosporins: A Kinetic and Molecular Modelling Analysis. J. Mol. Biol. 244, 625–639 (1994). 7990143 10.1006/jmbi.1994.1756

[b42] Chen C. C. H. & Herzberg O. Relocation of the catalytic carboxylate group in class A β-lactamase: the structure and function of the mutant enzyme Glu166→Gln: Asn170→Asp. Protein Eng. 12, 573–579 (1999). 10436083 10.1093/protein/12.7.573

[b43] Guerrero-Olazarán M., Rodríguez-Blanco L., Carreon-Treviño J. G., Gallegos-López J. A. & Viader-Salvadó J. M. Expression of a Bacillus Phytase C Gene in Pichia pastoris and Properties of the Recombinant Enzyme. Appl. Environ. Microbiol. 76, 5601–5608 (2010). 20601512 10.1128/AEM.00762-10 PMC2918954

[b44] Savitzky A. & Golay M. J. E. Smoothing and Differentiation of Data by Simplified Least Squares Procedures. Anal. Chem. 36, 1627–1639 (1964).

[b45] Stec B., Holtz K. M., Wojciechowski C. L. & Kantrowitz E. R. Structure of the wild-type TEM-1 beta-lactamase at 1.55 A and the mutant enzyme Ser70Ala at 2.1 A suggest the mode of noncovalent catalysis for the mutant enzyme. Acta Crystallogr. D Biol. Crystallogr. 61, 1072–1079 (2005). 16041072 10.1107/S0907444905014356

