# Supplementary material for: SpyRing interrogation: analyzing how enzyme resilience can be achieved with phytase and distinct cyclization chemistries
Source: Sci Rep. 2016 Feb 10;6:21151. doi: 10.1038/srep21151 (PMC4748275; doi:10.1038/srep21151)
Supplement: Supplementary Information [file srep21151-s1.pdf]

Supplementary Figures

SpyRing interrogation: analyzing how enzyme resilience can be achieved with phytase and distinct cyclization chemistries

Christopher Schoene[1], S. Paul Bennett[2] & Mark Howarth[1]
[1]Department of Biochemistry, University of Oxford, South Parks Road, Oxford, OX1 3QU, UK, [2]Sekisui Diagnostics UK Ltd., Operations Building, Liphook Way, Allington, Maidstone, Kent, ME16 0LQ, UK.

Correspondence and requests for materials should be addressed to M.H. (mark.howarth@bioch.ox.ac.uk)

Figure S1

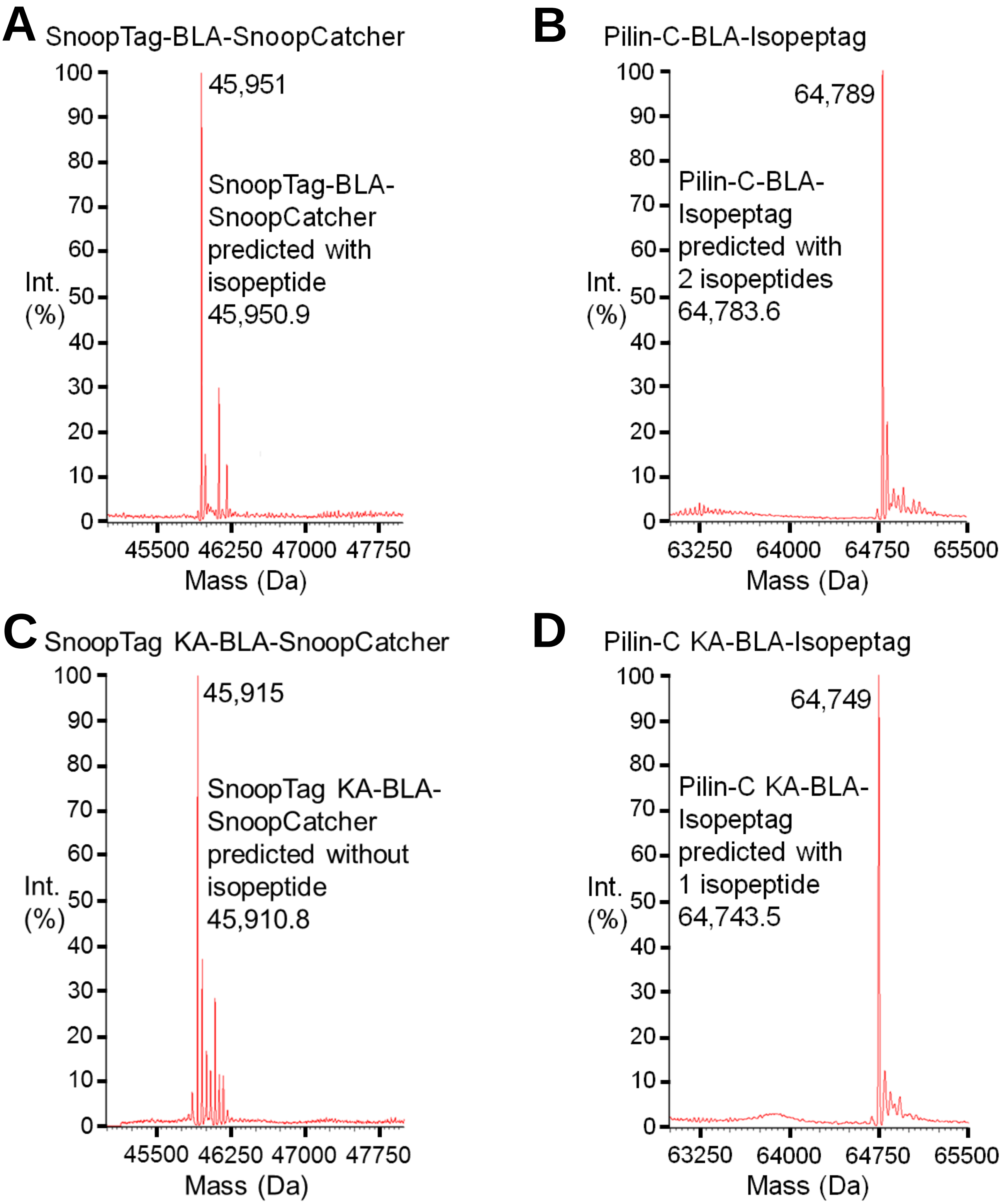

Mass spectrometry testing of SnoopRing and PilinRing cyclization. Electrospray MS of (A) SnoopTag-BLA-SnoopCatcher, (B) Pilin-C-BLA-Isopeptag, (C) SnoopTag KA-BLA-SnoopCatcher, and (D) Pilin-C KA-BLA-Isopeptag. In each case the observed mass of the principal peak is marked and the predicted mass is given.

Figure S2

A

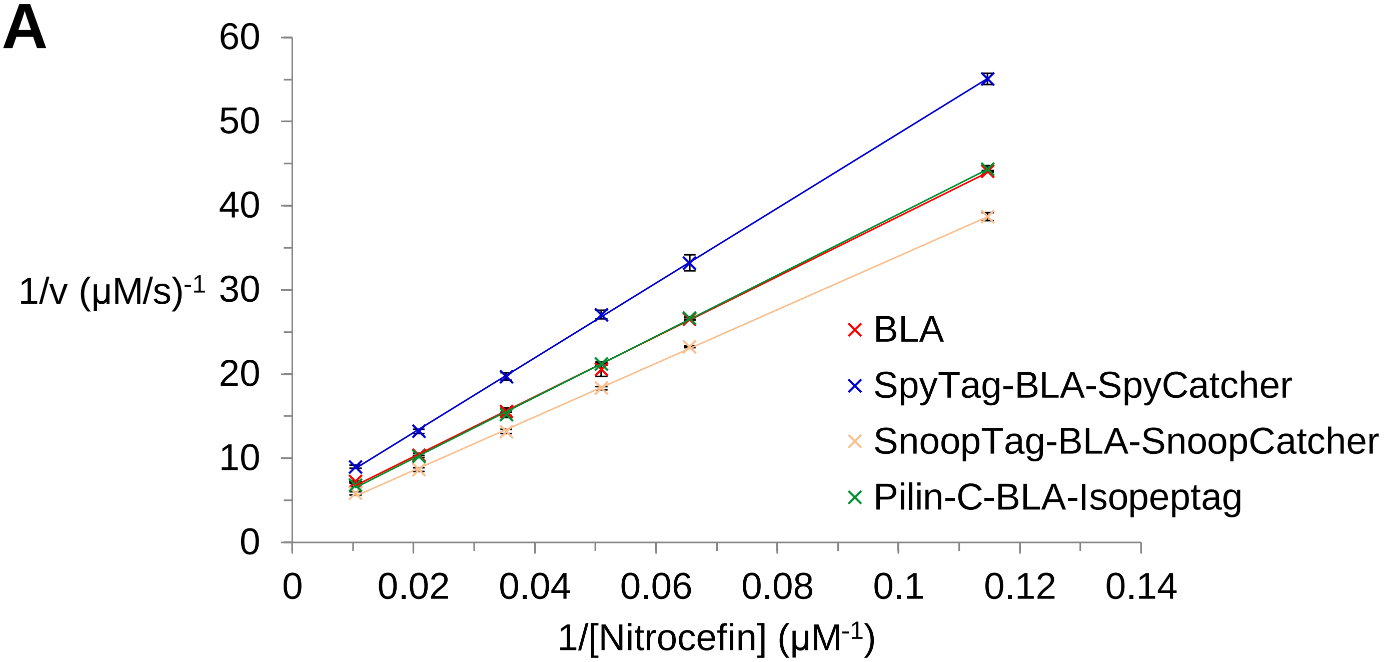

B

Construct	V_{max} (μM/s)	K_M (μM)	k_{cat} (s^{-1})	k_{cat}/K_M ($\mu M^{-1}s^{-1}$)
BLA	0.33 ± 0.008	120 ± 3	1200 ± 30	11 ± 0.02
SpyTag-BLA-SpyCatcher	0.24 ± 0.01	110 ± 6	910 ± 40	8.5 ± 0.1
SnoopTag-BLA-SnoopCatcher	0.46 ± 0.03	150 ± 10	1700 ± 100	12 ± 0.2
Pilin-C-BLA-Isopeptag	0.37 ± 0.01	130 ± 4	1400 ± 40	10 ± 0.08

Cyclization did not substantially affect the kinetic parameters of BLA. (A) Lineweaver-Burk plot of the PilinRing, SnoopRing, SpyRing and wild-type BLA constructs. 0.25 nM enzyme was incubated with different concentrations of nitrocefin in 50 mM NaH_2PO_4 pH 7.0 at 30 °C (mean of triplicate ± 1 s.d.; some error bars are too small to be visible). (B) Kinetic parameters of PilinRing, SnoopRing, SpyRing and wild-type BLA constructs for nitrocefin (mean ± 1 s.d. based on triplicate measurements).

Figure S3

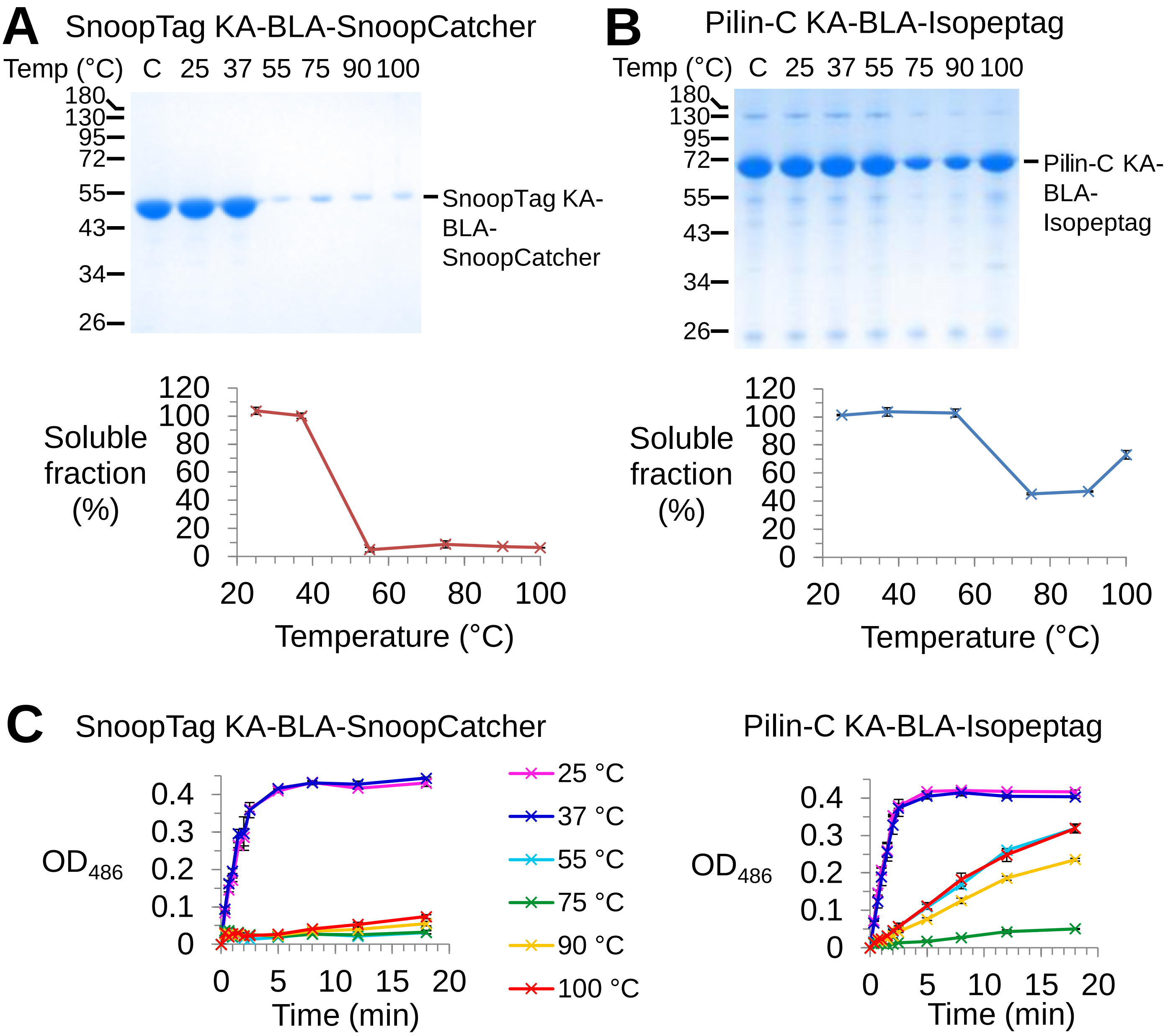

Point mutant controls of SnoopRing and PilinRing had less thermal resilience than the cyclized forms. (A) Aggregation resistance of SnoopTag KA-BLA-SnoopCatcher. The sample was heated at the indicated temperature for 10 min, centrifuged and the supernatant analyzed by SDS-PAGE with Coomassie staining. C is control without incubation. Triplicate data from SDS-PAGE were then plotted. (B) Aggregation resistance of Pilin-C KA-BLA-Isopeptag, tested as in (A). (C) Recovered catalytic activity of SnoopTag KA-BLA-SnoopCatcher and Pilin-C KA-BLA-Isopeptag. Proteins were incubated for 10 min at the indicated temp., before returning to RT and then running a colorimetric activity assay. (All are mean of triplicate ± 1 s.d.; some error bars are too small to be visible.)

Figure S4

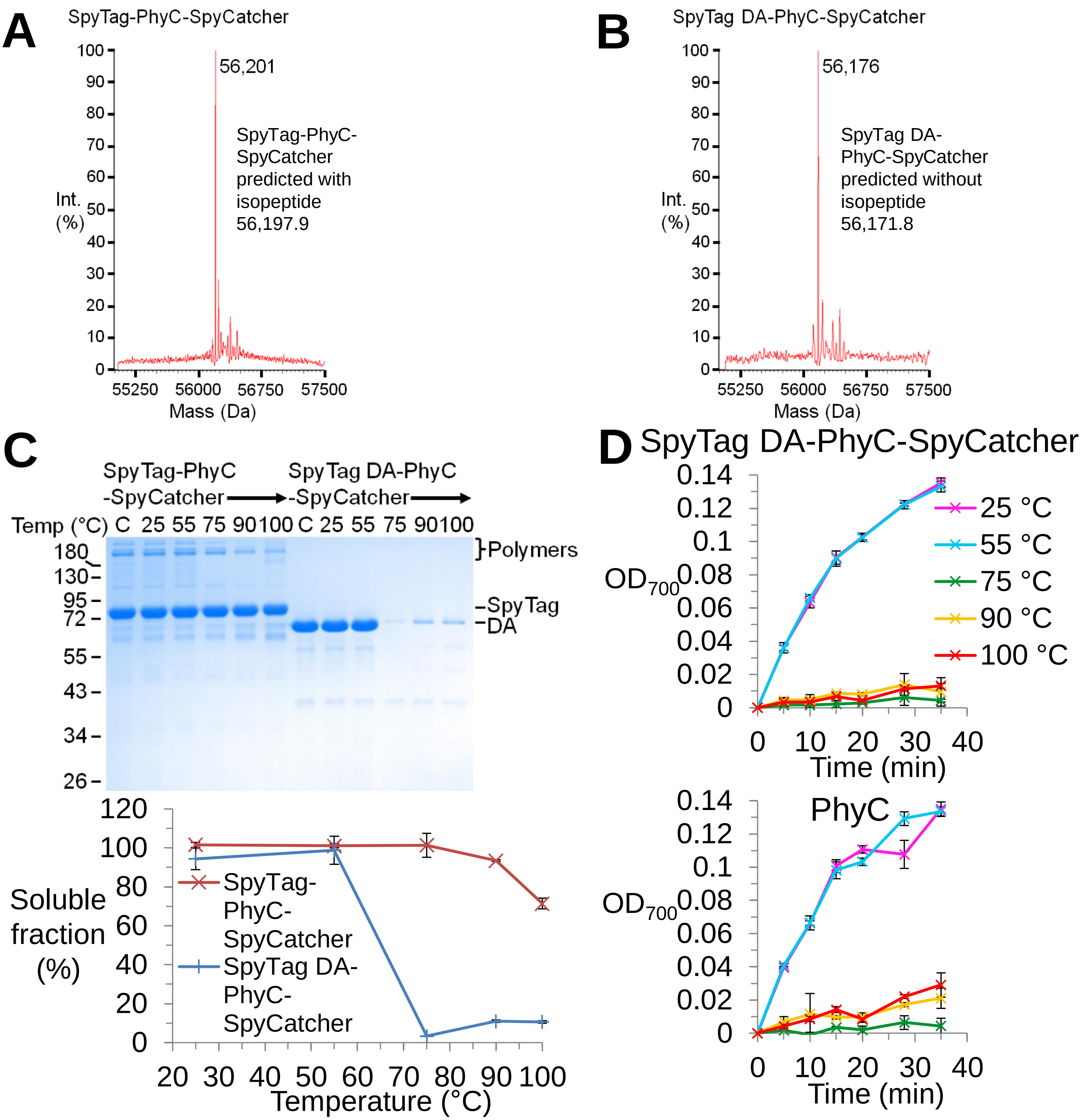

SpyRing cyclization of phytase analyzed by MS and by comparison to point mutant control. (A) MS of SpyTag-PhyC-SpyCatcher confirms isopeptide formation. (B) MS of SpyTag DA-PhyC-SpyCatcher showed no isopeptide formation. (C) SpyTag DA-PhyC-SpyCatcher had little aggregation-resistance. SpyTag-PhyC-SpyCatcher and SpyTag DA-PhyC-SpyCatcher were heated at the indicated temp. for 10 min, centrifuged and the supernatant analyzed by SDS-PAGE with Coomassie staining. C is control without incubation. (D) Catalytic activity of PhyC and SpyTag DA-PhyC-SpyCatcher was not resilient to heating. Proteins were incubated for 10 min at the indicated temp., before returning to RT and running a colorimetric activity assay for phosphate release from phytic acid. (All are mean of triplicate ± 1 s.d.; some error bars are too small to be visible.)

Figure S5

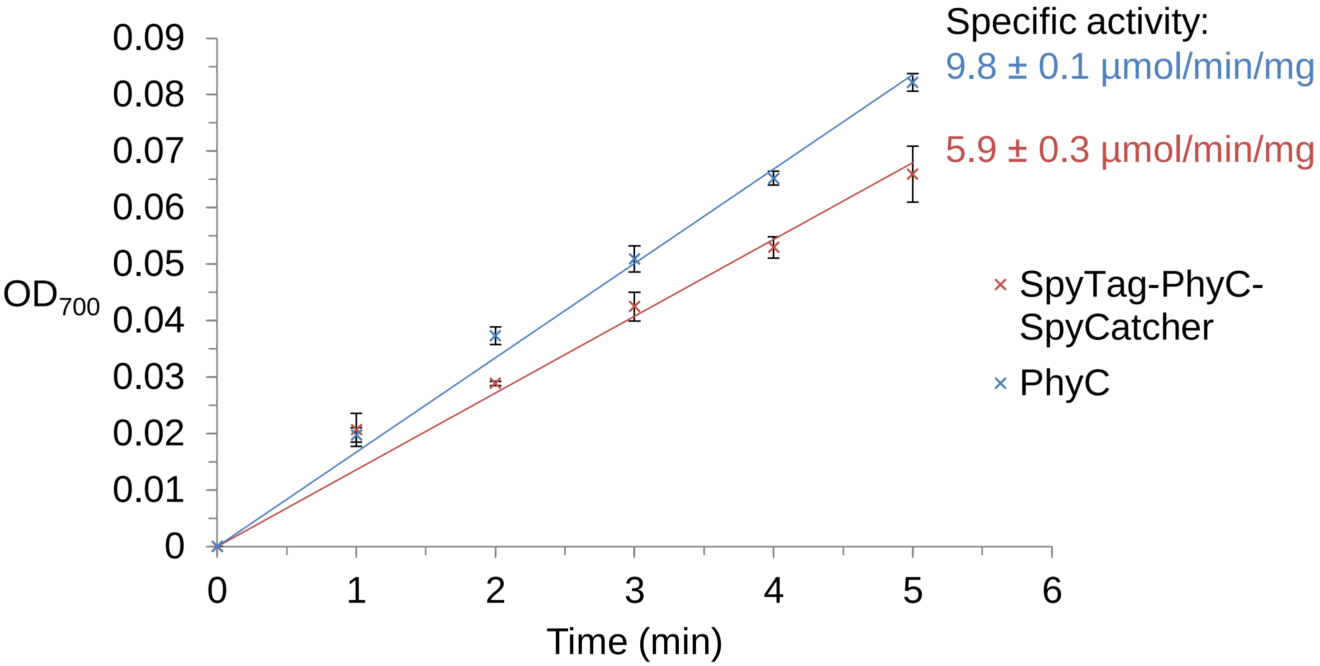

Cyclization did not substantially affect the specific activity of phytase. 40 nM PhyC or SpyTag-PhyC-SpyCatcher was incubated with 1.6 mM phytic acid for 1-5 minutes at 37 °C. Phosphate release was determined from OD_{700} using the molybdenum blue method with reference to a standard curve and the calculated specific activity is marked (mean of triplicate ± 1 s.d.; some error bars are too small to be visible).

Figure S6

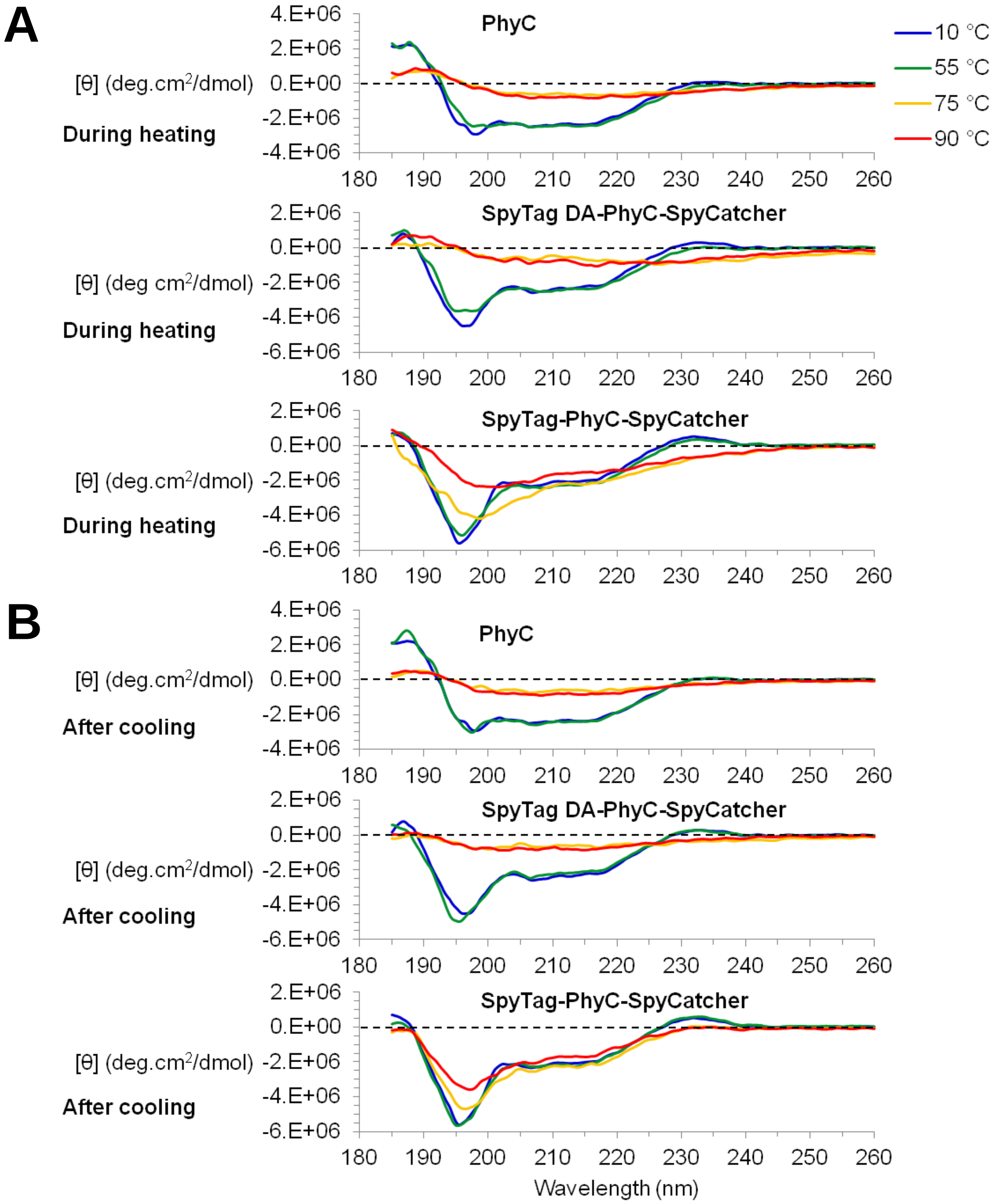

Analysis of SpyRing phytase secondary structure with heating. (A) Far-UV CD spectra of PhyC, SpyTag DA-PhyC-SpyCatcher and SpyTag-PhyC-SpyCatcher while incubated at the indicated temperature. Measurement was started after incubating at the indicated temperature for 3 minutes. (B) PhyC, SpyTag DA-PhyC-SpyCatcher and SpyTag-PhyC-SpyCatcher were incubated at the indicated temperature for 10 min, before cooling to 10 °C. After 3 min at 10 °C, the far-UV CD spectrum was acquired at 10 °C.

Figure S7

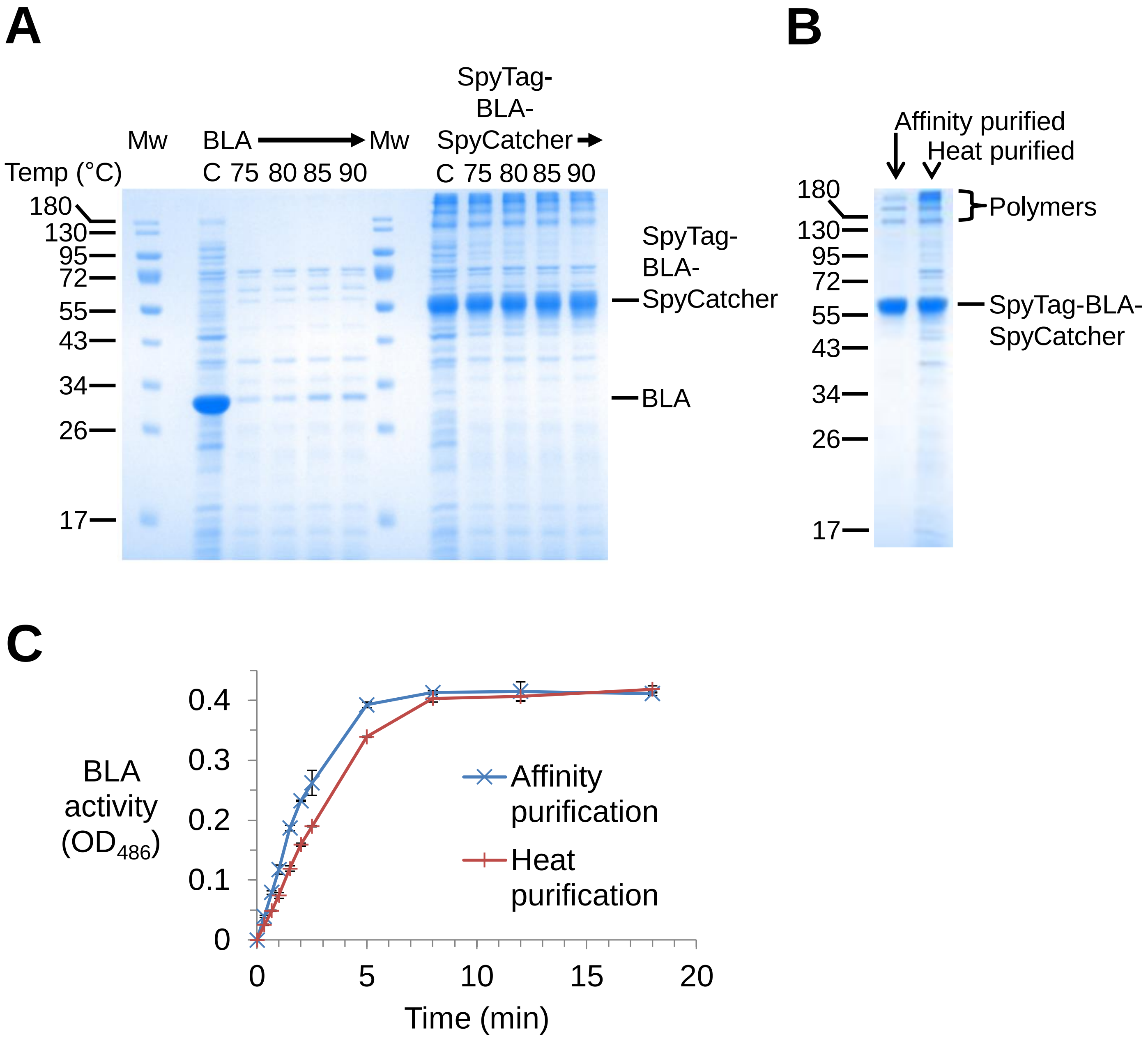

SpyRing cyclization of BLA allowed enzyme purification just by heating. (A) Heat-based purification of BLA. *E. coli* lysate expressing BLA or SpyTag-BLA-SpyCatcher was heated for 10 min at the indicated temperature, centrifuged, and the supernatant analyzed by SDS-PAGE with Coomassie staining. C is control without incubation. Mw are the molecular weight markers. (B) Comparing purity between heat-purified and affinity-purified BLA. SpyTag-BLA-SpyCatcher purified by Ni-NTA or heat-purification was boiled in SDS-loading buffer and analyzed by SDS-PAGE with Coomassie staining. (C) Heat purified BLA was still active. Catalytic activity of SpyTag-BLA-SpyCatcher purified by either affinity chromatography or by heat purification, from colorimetric nitrocefin assay. (All are mean of triplicate ± 1 s.d.; some error bars are too small to be visible.)
